# O07 - Phenotypes of atopic dermatitis depending on the timing of onset and the evolution in childhood

**DOI:** 10.1186/2045-7022-4-S1-O7

**Published:** 2014-03-14

**Authors:** Caroline Roduit, Audrey Dunn Galvin, Erika von Mutius, Juha Pekkanen, Jonathan B O’Hourihane, Josef Riedler, Charlotte Braun-Fahrländer, Roger Lauener

**Affiliations:** 1 University Children's Hospital Zurich and Christine Kühne-Center for Allergy Research and Education, Zurich, Switzerland; 2 Swiss Institute of Allergy and Asthma Research (SIAF), University of Zurich and Christine Kühne-Center for Allergy Research and Education, Zurich, Switzerland; 3 University Children’s Hospital Munich, Munich, Germany; 4 Department of Environment Health, National Institute for Health and Welfare, Kuopio, Finland; 5 Department of Respiratory Disease, UMR/CNRS 6249 Chrono-environnement, University Hospital of Besançon, Besançon, France; 6 Children’s Hospital Schwarzach, Schwarzach, Austria; 7 Swiss Tropical and Public Health Institute, Basel, Switzerland; 8 University of Basel, Basel, Switzerland; 9 Children’s Hospital St Gallen and Christine Kühne-Center for Allergy Research and Education, Davos, Switzerland

## Background

Atopic dermatitis is an inflammatory, pruritic skin disease that often occurs in early infancy with a chronic course. However, the description of different subtypes of atopic dermatitis, depending on the timing of onset and the evolution of the disease in early childhood, is lacking.

## Objective

To identify different phenotypes of atopic dermatitis, using the symptoms in the first 6 years of life from a prospective study, and whether they differ in their association with parental allergic status and prenatal farm-related exposures.

## Method

1045 children who participated in the birth cohort study, Protection Against Allergy-Study in Rural Environments (PASTURE), were included in the current study. Symptoms of atopic dermatitis were reported by parents from birth to 6 years of age by yearly questionnaires and defined as an intermittent or persistent itchy rash on typical locations. We used longitudinal latent class analysis (LCA) to identify different phenotypes of atopic dermatitis symptoms in childhood based on the first 6 years of life.

## Results

The LCA model with the best fit to PASTURE data was a model with 4 classes. Therefore, we could determine 4 phenotypes of atopic dermatitis symptoms, defined as follow: never or infrequent (894, 85.6 %), early-transient (52, 5.0 %), early-persistent (55, 5.3%), and late (44, 4.2%). The parental history of allergies was strongly associated with the early-persistent phenotype. Maternal contact to pets (cat or dog) during pregnancy showed a significantly protective effect only on the early-persistent phenotype. A same tendency was observed with prenatal contact to farm animals, even though not significant. However, maternal consumption of farm milk during pregnancy showed a protective effect, only on the early-transient phenotype of atopic dermatitis symptoms.

## Conclusion

Using latent class analysis, 4 different phenotypes of atopic dermatitis symptoms were identified. The association between prenatal exposures and atopic dermatitis symptoms were different depending on the phenotypes of atopic dermatitis.

